# Root-endophytic *Chaetomium cupreum* chemically enhances aluminium tolerance in *Miscanthus sinensis* via increasing the aluminium detoxicants, chlorogenic acid and oosporein

**DOI:** 10.1371/journal.pone.0212644

**Published:** 2019-02-22

**Authors:** Toshikatsu Haruma, Keiko Yamaji, Kazuyoshi Ogawa, Hayato Masuya, Yurina Sekine, Naofumi Kozai

**Affiliations:** 1 Graduate School of Life and Environmental Sciences, University of Tsukuba, Tsukuba, Ibaraki, Japan; 2 Forestry and Forest Products Research Institute, Tsukuba, Ibaraki, Japan; 3 Materials Sciences Research Center, Japan Atomic Energy Agency, Tokaimura, Ibaraki, Japan; 4 Advanced Science Research Center, Japan Atomic Energy Agency, Tokaimura, Ibaraki, Japan; Central Agricultural University, INDIA

## Abstract

*Miscanthus sinensis* Andersson is a pioneer plant species that grows naturally at mining sites. *Miscanthus sinensis* can detoxify aluminium (Al) by producing phytosiderophores, such as chlorogenic acid, citric acid, and malic acid, and localizing Al in cell walls. Root-endophytic *Chaetomium cupreum*, which produces microbial siderophores, enhances Al tolerance in *M*. *sinensis*. However, we could not determine whether the siderophores produced by *C*. *cupreum* actually enhance Al tolerance in *M*. *sinensis*, because the microbial siderophores have not yet been identified in previous research. The purpose of this study was to clarify how *C*. *cupreum* chemically increases Al tolerance in *M*. *sinensis* under acidic mining site conditions, especially considering siderophores. Using instrumental analyses, the siderophore produced by *C*. *cupreum* was identified as oosporein. Comparison of the stability constant between Al and phytosiderophores and oosporein indicated that oosporein could detoxify Al similarly to chlorogenic acid, which shows higher stability constant than citric acid and malic acid. Inoculation test of *C*. *cupreum* onto *M*. *sinensis* in acidic mine soil showed that *C*. *cupreum* promoted seedling growth, and enhanced Al tolerance via inducing chlorogenic-acid production and producing oosporein. These results suggested that *C*. *cupreum* could chemically enhance Al tolerance and might promote growth via reducing excessive Al in cell walls, the main site of Al accumulation. In addition, the chemical enhancement of Al tolerance by *C*. *cupreum* might be important for *M*. *sinensis* to adapt to acidic mining sites.

## Introduction

Acidification of ecosystems has become a global concern since the late 1970s, because industrial equipment and smelters emitted SO_x_ and NO_x_, which then caused acid rain [1]. Acidic soils promote the solubilization of aluminium (Al, the most abundant metal in the earth’s crust) and the phytotoxic Al ion is the main limiting factor for plant growth in acidic soils [2–5]. By contrast, plants have adapted to survive metal stress [6]. *Miscanthus sinensis* is a pioneer species in highly acidic soils [7] as well as acidic mining sites in Japan [8], and would be an appropriate plant species for the initial greening of acidic mining sites. Indeed, *M*. *sinensis* growing in acidic mining sites shows Al tolerance and accumulates high concentrations of Al compared with heavy metals in its root cells [9]. The Al tolerance mechanism of *M*. *sinensis* acts via the production of Al detoxicants (chlorogenic acid, malic acid, and citric acid) and the localization of Al into cell walls [9].

Recently, we became aware that root endophytes could be important for plants to survive under severe environments caused by salt, drought, herbivores, pathogens, and metals [10]. Root fungal endophytes increase the harmful-metal tolerance of plants by enhancing plant growth [11–14], and localizing harmful metals in cell walls [15, 16]. From a chemical aspect, root bacterial endophytes increase harmful metal tolerance of host plants via producing microbial siderophores [17, 18], which are defined as relatively low-molecular weight compounds capable of chelating Fe [19] and various metals, including Al [10, 20–22]. Siderophores could chelate metals to form a complex that is less toxic to the plant cells. Root endophytes, such as fungi and bacteria, producing microbial siderophores, would be also important to protect plants and microbes from metal toxicity [10, 23, 24]. In addition, plants also produce phytosiderophores, which detoxify harmful metals, including Al [20, 25], in their cells. Several root endophytes are known to induce host plants to produce secondary metabolites [26, 27]; therefore, root endophytes might enhance metal stress tolerance of plants by increasing phytosiderophores production in plant cells.

There are many kinds of microbial siderophores [28, 29]. To evaluate accurately the metal-detoxification activity of siderophores, the stability constants between Al and siderophores should be measured, because siderophores with higher stability constants with Al chelate Al more strongly compared with those with lower stability constants [30]. Thus, siderophores with higher stability constants could detoxify Al more efficiently. For example, the stability constants between Al and phytosiderophores, such as chlorogenic acid, citric acid, and malic acid produced by *M*. *sinensis* roots [9], are 15.1, 8.0, and 5.4, respectively [30, 31]; therefore, chlorogenic acid would be the best Al-detoxicant among them. Moreover, the amount of Al detoxified by siderophores could be calculated from amount of siderophores and the coupling ratio of Al to the siderophores. Thus, comparisons of the stability constants, together with the coupling ratio of Al between phytosiderophores and microbial siderophores, would provide important clues to evaluate the chemical contribution of root endophytes to Al stress tolerance in plants. Our previous study clarified that root-endophytic *Chaetomium cupreum*, which produced siderophores, enhanced Al stress tolerance in *M*. *sinensis* by accumulating Al in mycelia around the roots [9]. However, we could not determine whether the siderophores produced by *C*. *cupreum* actually enhanced Al tolerance in the plants, because we did not identify the siderophore produced by *C*. *cupreum* in previous research.

In the present study, we focused on the microbial siderophore(s) produced by root endophytic *C*. *cupreum*. The aims of this study were: 1) To identify the siderophore(s) produced by root-endophytic *C*. *cupreum*; and 2) to measure the coupling ratio and stability constant between Al and the identified siderophore. Finally, through *in vitro* inoculation of *C*. *cupreum* onto sterile *M*. *sinensis* seedlings, the amount of Al detoxified with microbial siderophores and phytosiderophores was calculated using the coupling ratio of Al to each siderophore. Furthermore, we quantified the microbial siderophore and phytosiderophore, especially, chlorogenic acid, which was detected at high levels and could chelate Al with a higher stability constant than citric acid and malic acid. We also discussed how root-endophytic *C*. *cupreum* would increase Al tolerance in *M*. *sinensis* in acidic mine soil.

## Materials and methods

### Ethics statement

The study site belongs to the Japanese National Forest. Our fieldwork, including collections of plant materials and soil, were permitted by the Ibaraki District Forest Office. Our investigations did not involve any endangered or protected species.

### Fungus and culture condition

*Chaetomium cupreum* (5R-7, NBRC111720), which was previously isolated from the roots of *M*. *sinensis* growing naturally in an acidic old mine site and identified by DNA analysis [9], was used in this study. Previously, we confirmed that *C*. *cupreum* produced siderophore(s) using the chrome azurol S (CAS)-Al assay [9]. *Chaetomium cupreum* was grown on 1% malt extract agar (1% MA, pH 5.5) for seven days at 23°C in the dark to obtain the edge of the mycelium before *C*. *cupreum* mycelium reached the edge of the petri dish. Three mycelial disks (5.5 mm i.d.) on the edge of the mycelium were inoculated into a 50-mL Erlenmeyer flask containing 30 mL of 1% malt extract (1% ME) liquid medium. The medium was incubated with shaking at 23°C in the dark for 3, 6, 9, 12, 15, or 18 days with three replications per incubation time. At each time point, the culture was filtered using no. 6 filter paper (Advantec, Tokyo, Japan), and rinsed with deionized water. The filter paper with the mycelia was dried at 80°C for 4 h, and dry weight (DW) of the mycelia was calculated by subtracting the DW of the empty filter paper from DW of the filter paper with the mycelia. The culture filtrate was used for pH measurement and detection of siderophores on CAS-Al agar medium. CAS-Al agar medium was prepared using the procedure for preparing CAS-Fe agar medium described in reference [32], except that AlCl_3_·6H_2_O was used instead of FeCl_3_·6H_2_O. The culture filtrate was sterilized through a 0.22-μm membrane filter (Waters, Milford, MA, USA) and the filtrate (250 μL) was aseptically injected into a sterile stainless steel-cylinder (10 × 6 mm i.d.) on CAS-Al agar medium. After 24 h of incubation in the dark at 23°C, the clear zone (mm) was measured to evaluate the Al-chelating activity. Each measurement was conducted three times and the values were averaged. Finally, the incubation time for siderophore isolation was selected as 12 days, because the Al-chelation activity of the culture filtrate was highest at 12 days during the 18-day incubation period (S1 Fig).

### Isolation of siderophore(s) from the culture filtrate

*Chaetomium cupreum* was grown on 1% MA for seven days, and 20 mycelial disks (5.5 mm i.d.) cut from the edge of the mycelia were inoculated into a 300-mL Erlenmeyer flask containing 100 mL of 1% ME liquid medium. Ten Erlenmeyer flasks were incubated with shaking at 23°C in the dark for 12 days. The culture filtrate (900 mL, 7.27 g DW) was concentrated to 150 mL, and extracted three times with ethyl acetate (50 mL each). After drying over Na_2_SO_4_, followed by *in vacuo* at 40°C, the organic layer (237 mg), and the water layer (7.03 g) were obtained. Residuals in the organic layer were crystalized using cold ethanol and the crystalline powder (red, amorphous) was dried in a vacuum desiccator (175.7 mg). The mother liquid was dried *in vacuo* at 40°C (60 mg). The organic layer, water layer, crystalline powder, or mother liquid, which was separately dissolved in 10% methanol to be equivalent to 250 μL of culture filtrate, was evaluated on CAS-Al agar medium to confirm siderophore production, as described above. Blank (1% ME liquid medium shaken for 12 days) was also tested. The crystalline powder, which showed Al-chelating activity (S2 Fig), was used for spectroscopic analysis.

### Spectroscopic analysis of siderophore(s) in the crystalline powder

The crystalline-powder solution (1.5 μg/mL in 50% methanol; 5 μmol/L) (10 μL) was analyzed using high performance liquid chromatography/electrospray ionization-mass spectrometry (HPLC/ESI-MS; LC/MS-2020 series, Shimadzu, Kyoto, Japan) equipped with UV-VIS detector (SPD-20A; Shimadzu) at 280 nm. N_2_ gas was used as the nebulizer gas (N2Supplier 24F; System instruments, Tokyo Japan), and MS was operated in total ion count mode (scanning range, 50–500 m*/z*). The HPLC conditions were as follows: Column, Mightysil RP-18 MS (150 × 2.0 mm; Kanto, Tokyo, Japan); eluent, aq. 0.1% formic acid (solvent A) and 100% acetonitrile (solvent B); and flow rate, 0.2 mL⁄min at 40°C. The following gradient was used for the eluent system: 0–10 min, 70% A and 30% B; 10–20 min, 50% A and 50% B; and 20–40 min, 100% B. The crystalline powder (50 μmol/L in 50% methanol) was also analyzed to measure its accurate mass using HPLC/ESI-high definition mass spectrometry (HDMS) (UPLC-SYNAPT G2; Waters, Milford, MA, USA) using direct injection mode. The HPLC/ESI-HDMS conditions were as follows: Flow rate, 20 μL⁄min; detection time, 3 min; total injection volume of sample, 60 μL.

Siderophore(s) in the crystalline powder were identified using X-ray diffraction (XRD) with an X-ray diffractometer (Ultima IV; Rigaku, Tokyo, Japan) with Cu-*K*α radiation (λ = 0.15418 nm). The sample was mounted on a glass plate and optically centered on the diffractometer. The diffraction data were collected in 0.02° steps in the 2θ degree range from 10° to 60°, at 23°C. Rietveld analysis was performed using the RIETAN-FP software program [33].

### Measurement of the stability constant for the complex between Al and oosporein

#### Coupling ratio of Al to oosporein

The continuous variations method (Job’s method) was used to determine the coupling ratio of Al to oosporein. Three kinds of mixed solutions of AlCl_3_·6H_2_O (final concentration in deionized water; 1 mM) (Wako, Japan) and purified oosporein (final concentration in deionized water; 1, 2, or 3 mM) were prepared. The pH of each solution was adjusted to 6 using 0.1 M KOH (Wako). UV-VIS absorption spectra were obtained using a UV-VIS spectrophotometer (UV-2450; Shimadzu) using cells with a 1 cm path length.

#### Dissociation constant of oosporein, and the stability constant for the complex between Al and oosporein

To determine the dissociation constant, oosporein solution (3 mM dissolved in deionized water) was prepared with an ionic strength of 0.1 (KCl). Titration was performed at 25°C in a water-jacketed, thermostatically controlled glass titration flask. Oosporein solution was titrated with KOH (0.1 M, Wako) using a 10.000-mL precision burette (Miyahara Measuring Instruments, Osaka, Japan), and the pH was continuously determined using a pH meter (F-71; Horiba, Kyoto, Japan). Nitrogen gas was continuously maintained over the titrand surface during titration. A mixed solution containing AlCl_3_·6H_2_O (1 mM in deionized water) and oosporein (3 mM in deionized water) was also prepared, and titration was conducted as described above to determine the stability constant for the complex. The stability constant was calculated using Bjerrum’s method [34]. The titration curve for oosporein was shown in S3 Fig, which indicated that oosporein was ionized in two steps, when 1.325 mL and 2.650 mL of 1 M KOH were added, respectively. Oosporein, which possesses four protons, was shown as H_4_L. For the first ionization step, the formula was;

$$H_{4}L⇄2H+H_{2}L$$ (1)

where oosporein, which releases two protons, is shown as H_2_L. Charges were omitted. The concentrations were calculated as follows;

$$\left[H_{4}A]=C(1-\alpha_{d1}\right)$$ (2)

$$[H]=2C\alpha_{d1}$$ (3)

$$[H_{2}A]=C\alpha_{d1}$$ (4)

where *C* was the initial concentration of oosporein (= 0.003 M), and *α*_d1_ was degree of dissociation of oosporein. Using these values, dissociation constant at the first step (*K*_1_) could be expressed as follows;

$$K_{1}=\alpha_{d1}{\left[H\right]}^{2}/\left(1-\alpha_{d1}\right)$$ (5)

According to [35], *α*_d1_ was expressed as below;

$$\alpha_{d1}=\alpha_{n1}+(\left[H]-[OH\right])/2C$$ (6)

where *α*_n1_ (= 0.5) was degree of neutralization of oosporein. *α*_d1_ was calculated using the titration curve (S3 Fig). Finally, *K*_1_ was calculated as 3.98 × 10^−9^ using Eq (5). For the second ionization step, the formula was:

$$H_{2}L⇆2H+L$$ (7)

The concentrations and dissociation constant at the second step (*K*_2_) could be expressed as:

$$\left[H_{2}L]=C(1-\alpha_{d2}\right)$$ (8)

$$[H]=2C\alpha_{d2}$$ (9)

$$[L]=C\alpha_{d2}$$ (10)

$$K_{2}=\alpha_{d2}{\left[H\right]}^{2}/\left(1-\alpha_{d2}\right)$$ (11)

In the same way as *K*_1_, *K*_2_ was calculated as 5.30 × 10^−15^ using Eq (11). According to Bjerrum’s method, the formation function could be expressed as:

$$n=\left\{\left[L_{t}]-(\frac{{\left[H\right]}^{4}}{K_{1}K_{2}}+\frac{{\left[H\right]}^{2}}{K_{2}}+1)[L\right]\right\}/\left[{Al}_{t}\right]$$ (12)

where, n is the average number of ligands chelating an Al ion, and L_t_ and Al_t_ are the total oosporein and Al, respectively. [L] was expressed according to the following formula:

$$[L]=\left\{\left(4-a)[L_{t}]-[H]+[OH\right]\right\}/\left(\frac{4{\left[H\right]}^{4}}{K_{1}K_{2}}+\frac{2{\left[H\right]}^{2}}{K_{2}}\right)$$ (13)

where a was the ratio of titrated hydroxide ion to one molecule of oosporein. Finally, the formation curve was created.

### Inoculation test of *C*. *cupreum* onto *M*. *sinensis* seedlings

#### Sterile seedlings

*Miscanthus sinensis* seeds were collected at the mine site [9] in November 2016 and stored at 4°C before use. After the seeds were soaked in deionized water for 5 min, they were surface-sterilized using 70% ethanol for 2 min, 7.5% hydrogen peroxide solution for 5 min, and 70% ethanol for 2 min. The seeds were then rinsed twice with sterile deionized water for 5 min to remove reagents. The axenic seeds were incubated on 1/3 Hoagland medium containing 1.5% agar for 2 weeks (14 h light at 25°C/10 h dark at 20°C) in a growth chamber (NK Systems LP-100S, Nippon Medical & Instruments Co., Osaka, Japan) to prepare the same growth stage (second leaf stage) of *M*. *sinensis* seedlings for the inoculation test. Germination started after 3 days of incubation, and seedlings at the second leaf stage were used for the inoculation test.

#### Sterile root-zone soil

Root-zone soil used for the inoculation test was collected in November 2013 from the mine site [9]. The soil was dried and sieved (< 2 mm), and then sterilized using irradiation with 30-kGy γ-rays. After sterilization, the pH (H_2_O) was 4.23 ± 0.01, exchangeable Al was 445.12 ± 9.34 mg/kg, and exchangeable Cu, Pb, and Zn were 81.44 ± 1.39 mg/kg, 185.38 ± 0.74 mg/kg, and 14.72 ± 0.38 mg/kg, respectively [9]. On a clean bench, sterilized soil (30 g) was transferred into a sterilized glass pot (10 cm height × 5.5 cm i.d.) and sterile deionized water (30 mL) was added. The glass pots were used for the inoculation test.

#### Mycelial suspension

*Chaetomium cupreum* 5R-7 was used for the inoculation test. Twenty mycelial disks (5.5 mm i.d.) of *C*. *cupreum*, cut from the edge of the mycelia, were inoculated into a 300-mL Erlenmeyer flask containing 100 mL of 1% ME liquid medium with shaking at 23°C in the dark for 12 days. After incubation, the mycelia were rinsed with sterile deionized water to remove the medium. The mycelia were then homogenized using a homogenizer (HG-200, AS ONE Corporation, Osaka, Japan), and sterile deionized water was added to prepare a mycelial suspension containing 6 mg of mycelial DW per milliliter. Sterile deionized water was used as a control.

#### Growth condition in the inoculation test

Four sterile seedlings were aseptically transferred into a sterile glass-pot, and the mycelial suspension (400 μL) was inoculated close to the roots. Two conditions were prepared as follows: (1) Seedlings with *C*. *cupreum* and (2) seedlings with sterile deionized water as a control. Five replicated pots were prepared per condition. Twenty seedlings per condition were grown for 36 days (light: 14 h, 25°C /dark: 10 h, 20°C), and used for the following measurements; root length and fresh weight (FW) of the aboveground parts and roots. After measurement, one seedling was randomly selected from each pot (i.e., five replications per condition) and used for the following measurements; DW, water content, and elements concentrations of the aboveground parts and roots. The aboveground parts and roots were pyrolyzed in concentrated HNO_3_. Al, heavy metals (Cu, Fe, Pb and Zn), and inorganic elements (Ca, K, Mg and P) were analyzed using inductively coupled plasma optical emission spectrometry (Optima 7300 V, PerkinElmer, Waltham, MA, USA). Replication results were averaged, and the standard errors were calculated.

To check whether the inoculation test was successful, *C*. *cupreum* was re-isolated and trypan-blue-stained roots were observed microscopically. One seedling was randomly selected from each pot (i.e., five seedlings per condition) to re-isolate *C*. *cupreum*. After the roots were carefully washed with running water and deionized water, they were surface-sterilized with 70% ethanol for 1 min, followed by 3.75% H_2_O_2_ solution for 5 min and 70% ethanol for 1 min. The roots were then rinsed with sterilized deionized water to remove reagents, dried on sterile filter paper on a clean bench, and then cut into approximately 5 mm pieces using a sterile scalpel. The root pieces were put on 1% MA and incubated at 23°C in the dark for 14 days. For microscopic observation of fungal hyphae, another seedling was randomly selected from each pot (i.e., five seedlings per condition). Fungal hyphae were stained by trypan blue, and observed microscopically (CKX53, Olympus, Tokyo, Japan).

#### Quantification of siderophores in roots and calculation of Al detoxified with siderophores

One seedling was randomly selected from each pot (i.e., five seedlings per condition), and the roots were extracted with methanol at 23°C in the dark overnight. The methanol extract was filtered and dried *in vacuo* at 60°C. The dried extract was re-dissolved in 50% methanol (200 μL) and 10 μL of the extract was analyzed by HPLC according to a previous study [36] to quantify chlorogenic acid. Furthermore, oosporein, which is a microbial siderophore produced by *C*. *cupreum*, was quantified via HPLC/ESI-MS as described above, using the negative selected ion monitoring mode at 305 m*/z*.

To calculate the amount of Al detoxified by the siderophores, the water content (%) in control and inoculated roots (90.1 ± 0.7% and 87.5 ± 0.5%, respectively), quantification data, and coupling ratios (chlorogenic acid:Al = 1:1 [31]; oosporein:Al = 2:1) were used. The amounts of Al detoxified by the siderophores were shown as μmol/100 mg DW of roots.

### Statistical analysis

Statistical analysis was conducted using SPSS statistics software for Windows (ver. 24.0.0.0, IBM, Armonk, NY, USA). Differences in Al-chelating activities of the culture filtrate after separation were evaluated using one-factor ANOVA test (Tukey HDS). In the inoculation test, differences in seedling growth variables, concentrations; amounts of Al, heavy metals, and other inorganic elements; concentrations of siderophores; and amounts of Al detoxified by the siderophores were evaluated using Student’s *t*-test. Differences were considered significant at *P* < 0.05.

## Results

### Identification of siderophore(s) produced by *C*. *cupreum*

HPLC/ESI-MS analysis clarified that the molecular weight of the siderophore was *m/z* 305 ([M − H]^−^) and *m/z* 306 ([M]^−^). The molecular formula was elucidated to be C_14_H_10_O_8_ by HPLC/ESI-HDMS (found 305.0293, [M − H]^−^; calculated 305.0297).

**Fig 1:**
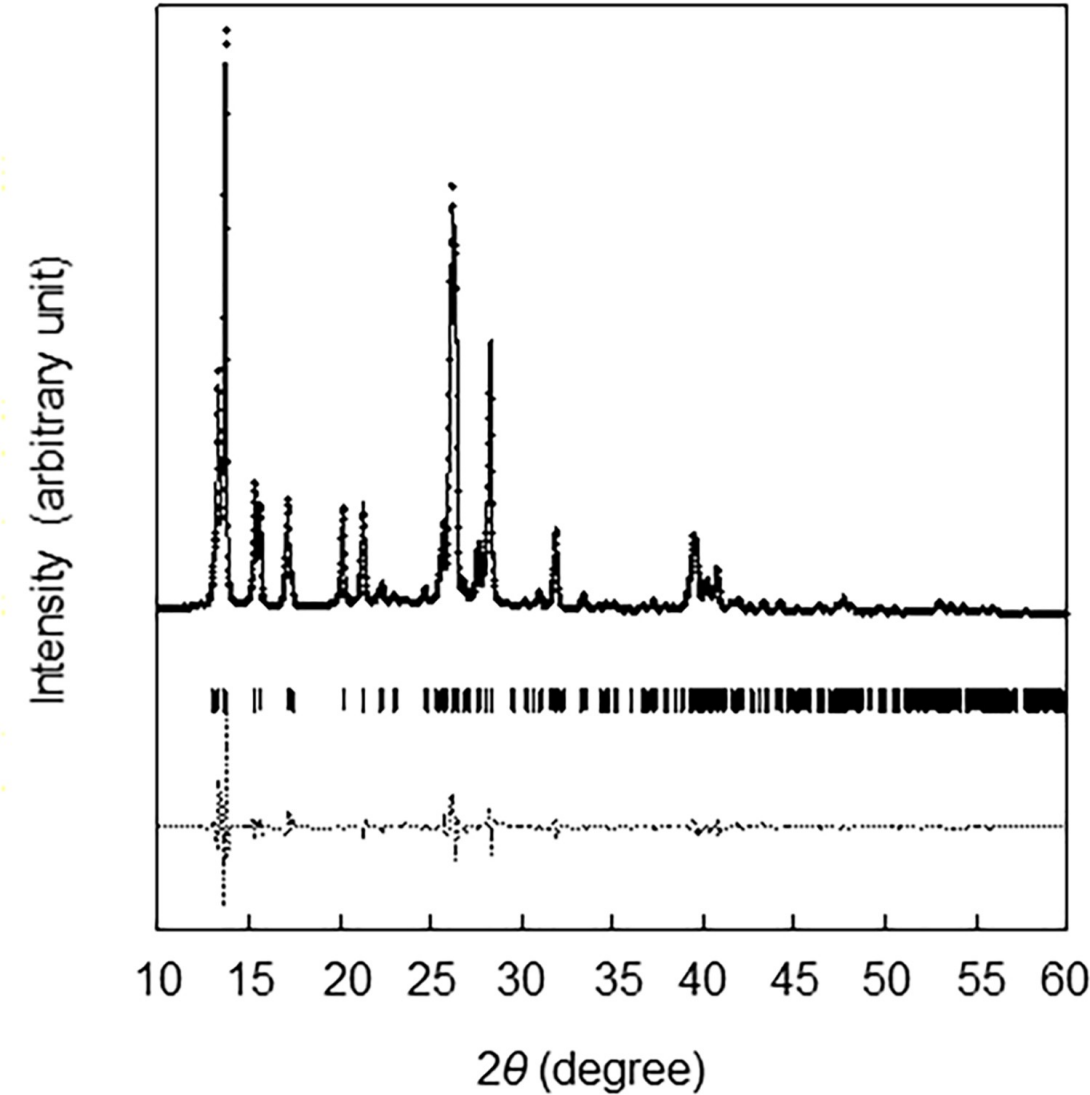
X-ray diffraction profile of the siderophore produced by *C*. *cupreum*. The closed black circles represent the measured intensity. The solid curve shows the calculated diffraction pattern using the best-fit parameters from Rietveld analysis. The peak positions calculated from the structure of C_14_H_10_O_8_ (C2/*c*) are shown by ticks below the diffraction patterns. The dashed lines are the deviation between the measured and calculated intensities.

**Fig 2:**
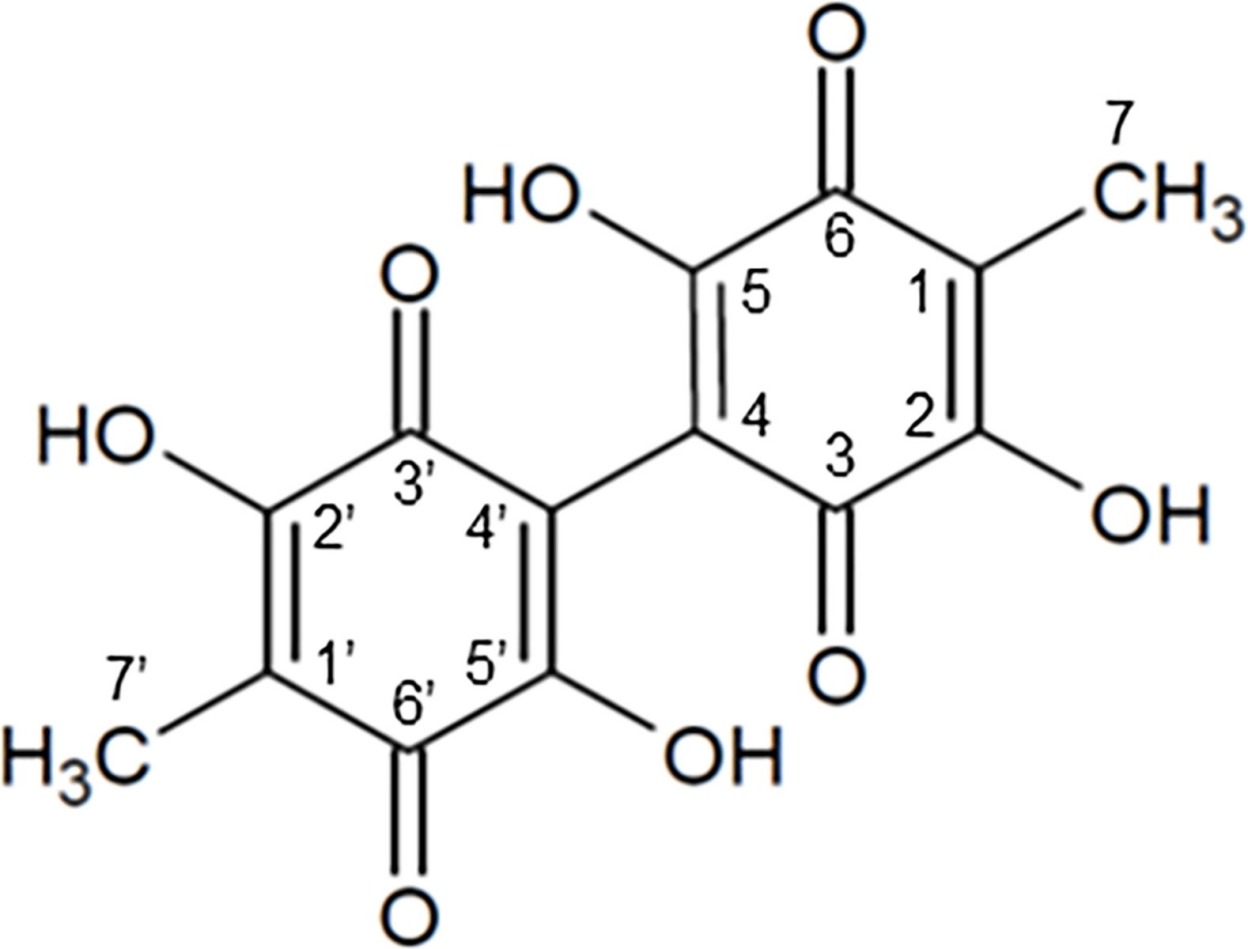
Chemical structure of oosporein. Carbons are numbered according to He et al. [37].

The XRD pattern of the sample is shown in Fig 1. The profile showed several sharp peaks and the feature of the peaks agreed with that of oosporein, C_14_H_10_O_8_, which has a monoclinic structure (C2/*c*) [37]. The results of Rietveld refinement for the profile, which are shown in Fig 1, and the structural parameters obtained by the analysis, are presented in Table 1. The results of XRD identified the siderophore produced by *C*. *cupreum* as oosporein. The structure of oosporein is shown in Fig 2.

**Table 1:** Lattice constants of the siderophore produced by *C*. *cupreum*.

*a* (nm)	*b* (nm)	*c* (nm)	*β* (°)	*V* (nm^3^)
1.197160 (92)	0.831636 (54)	1.377461 (86)	105.9730	1.3184553 (1558)

The *a*, *b*, and *c* represent the crystal-axes length, and *β* shows the angle between the crystal axes *a* and *c*. The unit-cell volume is shown as *V*.

### Stability constant for the complex between Al and oosporein

The UV-VIS absorption spectra of Al-oosporein chelates is shown in S4 Fig. Oosporein had two peaks at 211 nm and 298 nm in water at pH 6. The 1:1 molar ratio of oosporein to Al also had two peaks at 212 nm and 325 nm. The 2:1 molar ratio of oosporein to Al had a specific peak at 256 nm, in addition to two peaks at 217 nm and 317 nm. This result showed that oosporein chelated Al at a 2:1 molar ratio, and had a peak at 256 nm. The 3:1 molar ratio of oosporein to Al also had a peak at 255 nm besides the two peaks at 215 nm and 309 nm. If oosporein would chelate Al at a 3:1 molar ratio, the peak of Al-oosporein complex at the 3:1 molar ratio should be shifted to a shorter wavelength than 256 nm. Thus, our results indicated that oosporein chelated Al at a 2:1 molar ratio.

**Fig 3:**
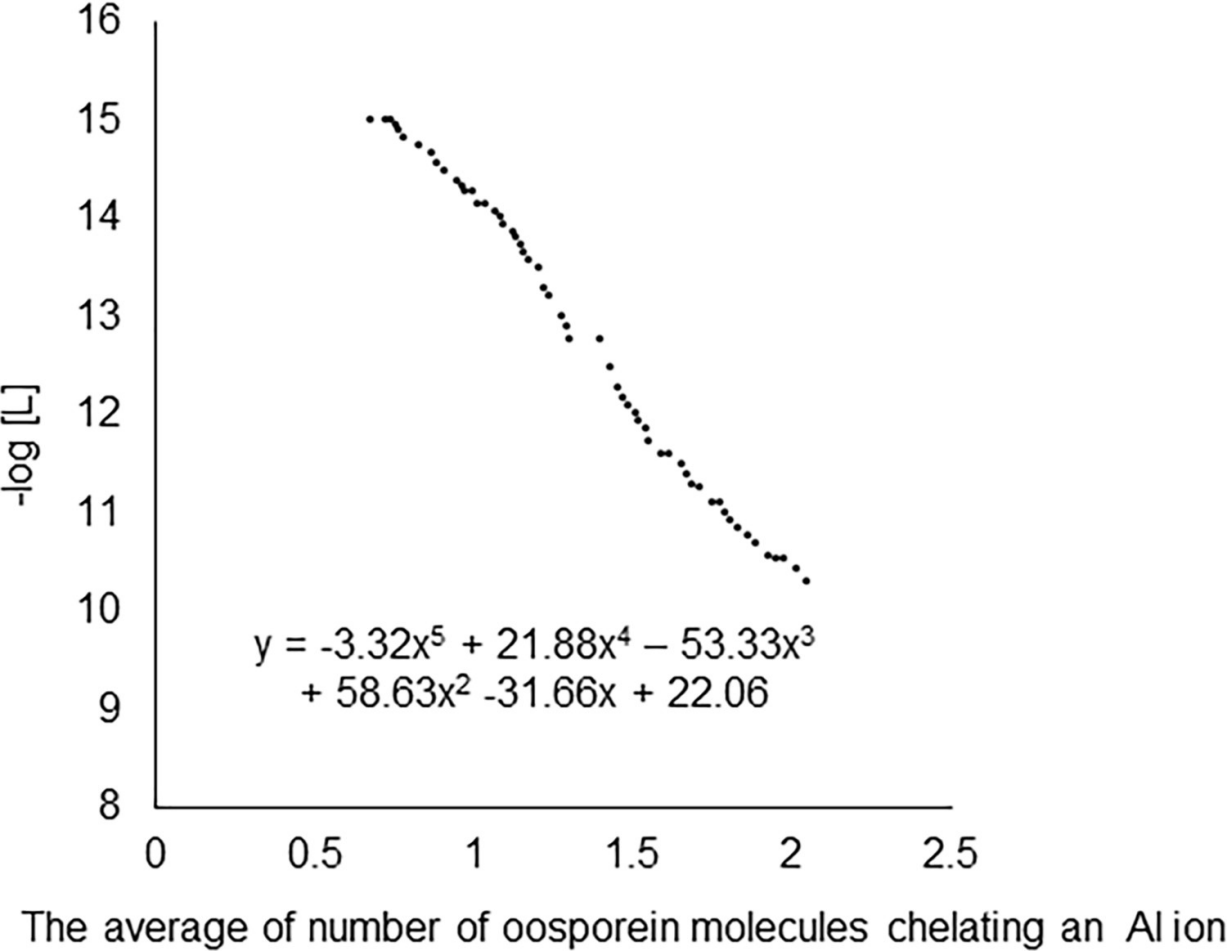
The formation curve of oosporein-Al complex. [L] is the concentration of oosporein that could not chelate Al. The formula shown is that for the approximate curve.

S3A Fig shows that oosporein was ionized in two steps at pH 4.21 and pH 7.14, and the dissociation constants of oosporein (*K*_1_ and *K*_2_) were calculated as 3.98 × 10^−9^ and 5.30 × 10^−15^, respectively, using Eq (1). According to Bjerrum’s method and the titration curve of Al and oosporein (S3B Fig), the formation curve was obtained (Fig 3). When oosporein chelates Al at a 2:1 molar ratio, and x in the equation shown in Fig 3 is 1.5 and the stability constant was calculated as 12.1 using the equation.

### Effect of *C*. *cupreum* on *M*. *sinensis* seedling growth and concentrations of Al and inorganic elements, and detection of oosporein and chlorogenic acid

After 36 days of incubation, the inoculation test of *M*. *sinensis* seedlings with *C*. *cupreum* was successfully performed without contamination, because *C*. *cupreum* alone was isolated from roots inoculated with *C*. *cupreum* and no microorganisms were isolated from the control roots. Although microsclerotia, which are infection structures formed by root endophytes, were not observed, mycelia growing along the epidermal cells were observed.

**Fig 4:**
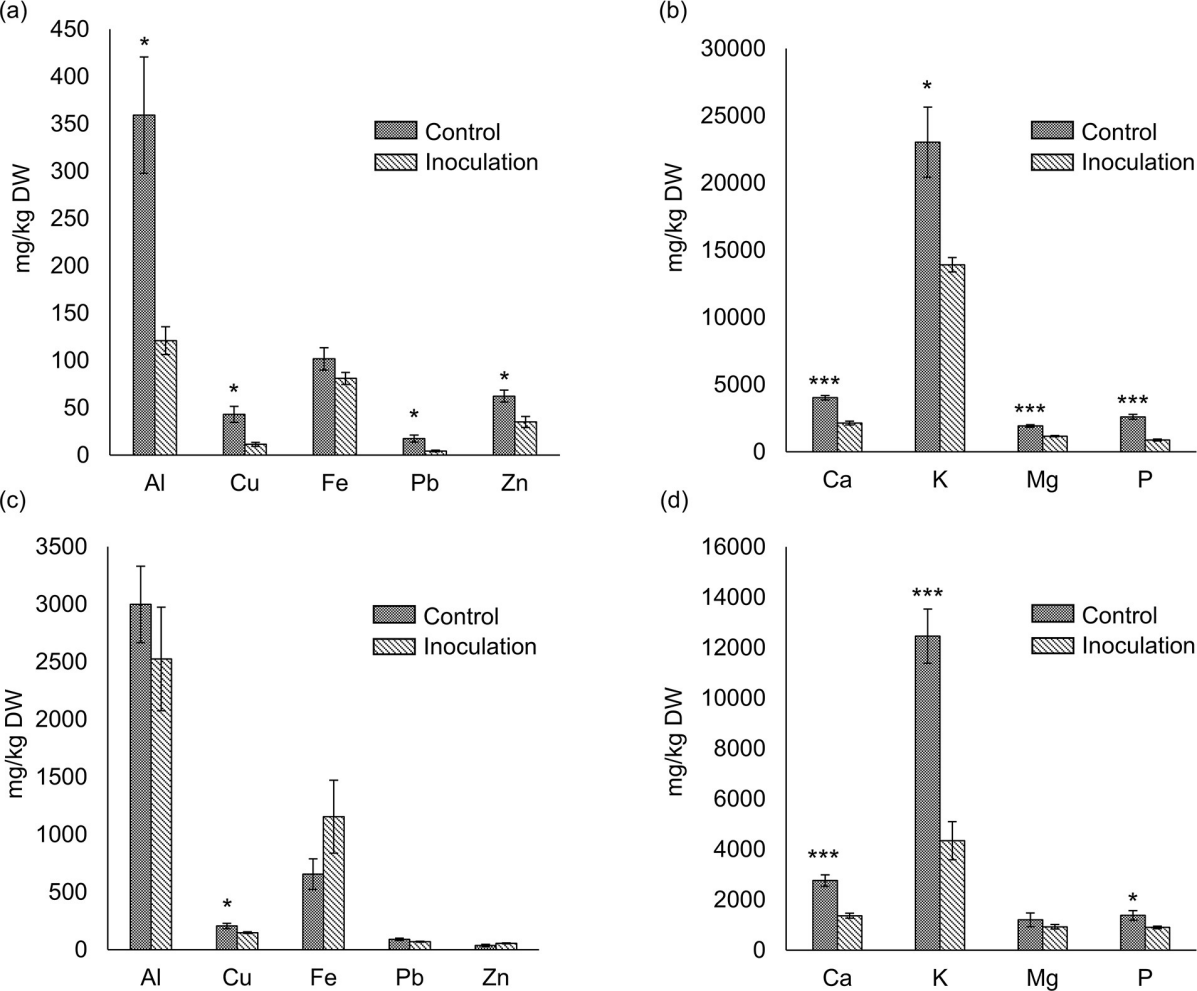
Concentrations of elements in *Miscanthus sinensis* seedlings in the inoculation test. (a) Concentrations of Al and heavy metals in the aboveground parts, and (b) concentrations of inorganic elements in the aboveground parts. (c) Concentrations of Al and heavy metals in the roots, and (d) concentrations of inorganic elements in the roots. Differences between seedlings inoculated with *C*. *cupreum* and control seedlings were evaluated using Student’s *t*-test. Results are expressed as means ± SE (n = 5). ***, *P* < 0.001; *, *P* < 0.05.

**Fig 5:**
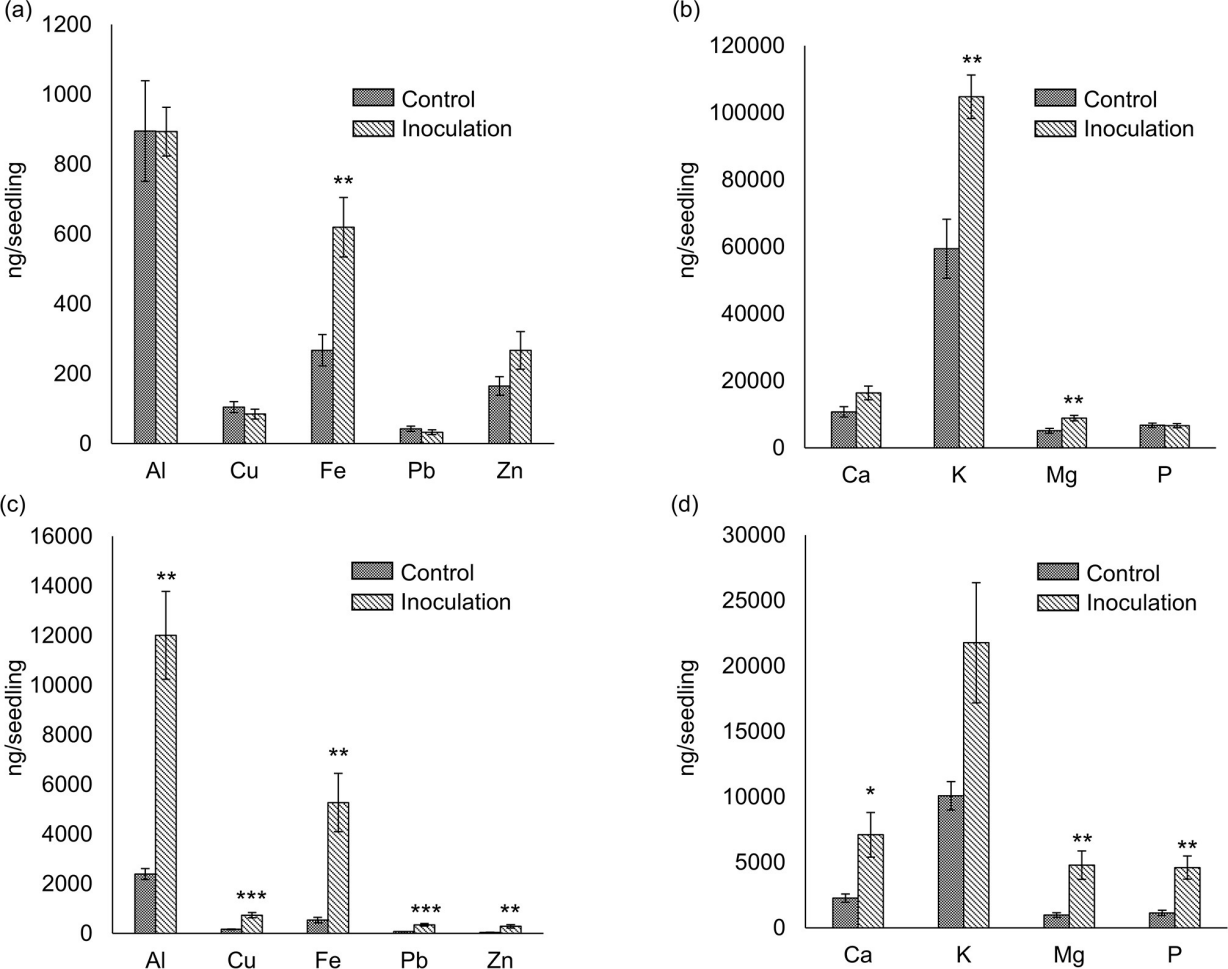
Amounts of elements in *Miscanthus sinensis* seedlings in the inoculation test. (a) Amounts of Al and heavy metals in aboveground parts of *M*. *sinensis*, and (b) amounts of inorganic elements in the aboveground parts. (c) Amounts of Al and heavy metals in *M*. *sinensis* roots, and (d) amounts of inorganic elements in the roots. Differences between seedlings inoculated with *C*. *cupreum* and control seedlings were evaluated using Student’s *t*-test. The results are expressed as means ± SE (n = 5). ***, *P* < 0.001; **, *P* < 0.01; *, *P* < 0.05.

Under acidic mine soil conditions, *C*. *cupreum* significantly enhanced *M*. *sinensis* seedling growth, such as root length (*P* < 0.001), FW of aboveground parts and roots (*P* < 0.001), DW of aboveground parts (*P* < 0.001), and DW of roots (*P* < 0.01) (Table 2 and S5 Fig). *Chaetomium cupreum* significantly decreased the concentrations of Al, Cu, Pb, Zn, K (*P* < 0.05), Ca, Mg, and P (*P* < 0.001) in the aboveground parts (Fig 4A and 4B), and Cu, P (*P* < 0.05), Ca, and K (*P* < 0.001) in the roots (Fig 4C and 4D). In contrast, *C*. *cupreum* significantly increased the amounts of Fe, K, and Mg (*P* < 0.01) in the aboveground parts (Fig 5A and 5B); and Cu, Pb (*P* < 0.001), Al, Fe, Zn, Mg, P (*P* < 0.01), and Ca (*P* < 0.05) in the roots (Fig 5C and 5D).

**Table 2:** Seedling growth in the inoculation test.

Treatment	Root length (cm)	Aboveground parts FW (mg)	Roots FW (mg)	Aboveground parts DW (mg)	Roots DW (mg)
Control	3.5 ± 0.4	7.9 ± 0.7	19.6 ± 2.2	0.8 ± 0.1	2.6 ± 0.3
Inoculated	36.3 ± 3.4***	31.5 ± 2.6***	30.0 ± 1.7***	5.1 ± 0.9***	7.6 ± 0.5**

FW, fresh weight; DW, dry weight. Results are expressed as means ± SE (n = 20 for root length and FW of aboveground parts and roots; n = 5 for DW of aboveground parts and roots). Differences between treatments were evaluated using Student’s *t*-test.

***, *P* < 0.001

**, *P* < 0.01.

**Fig 6:**
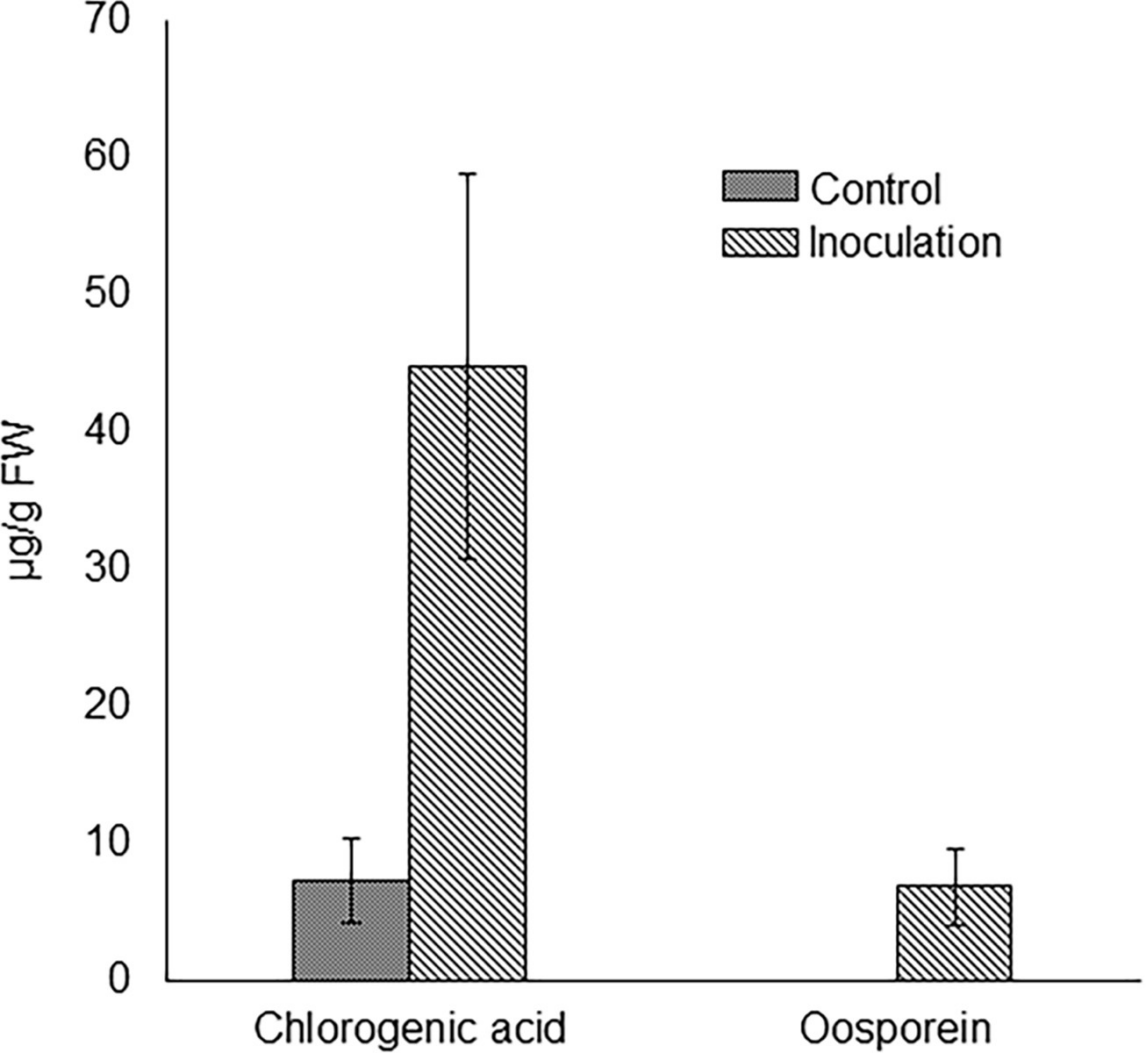
Concentrations of oosporein and chlorogenic acid in the inoculation test. Differences between treatments were evaluated using Student’s *t*-test. The results are expressed as means ± SE (n = 5). The concentration of chlorogenic acid was slightly higher in the roots inoculated with *C*. *cupreum* compared with that in the control roots (*P* = 0.051).

Oosporein was detected only from the roots inoculated with *C*. *cupreum*. In addition, chlorogenic acid, which could detoxify Al [31], was detected from control roots and the roots inoculated with *C*. *cupreum* (Fig 6). The concentration of chlorogenic acid was slightly higher in the roots inoculated with *C*. *cupreum* than in the control roots (Fig 6, *P* = 0.051). The total amounts of Al detoxified using the siderophores showed that the amount of detoxified-Al increased by approximately four times in the roots inoculated with *C*. *cupreum* than in the control roots (Table 3, *P* < 0.05).

**Table 3:** Siderophores contribution to Al detoxification in control roots and inoculated roots with *Chaetomium cupreum*.

Treatment	Chlorogenic acid (μmol/100 mg DW)	Oosporein (μmol/100 mg DW)	Total amount of Al detoxified using siderophores (μmol/100 mg DW)
Control roots	0.03 ± 0.01	ND	0.03 ± 0.01
Inoculated roots	0.10 ± 0.04	0.02 ± 0.01	0.11 ± 0.03*

DW, dry weight; ND, not detected. Results are expressed as means ± SE (n = 5). Differences between treatments were evaluated by Student’s *t*-test.

*, *P* < 0.05.

## Discussion

The siderophore produced by root-endophytic *C*. *cupreum* isolated from *M*. *sinensis*, which accumulates high concentrations of Al [9], was identified as oosporein. Oosporein isolated from an insect-pathogenic fungus *Beauveria bassiana* is an effective antibiotic compound against bacteria [38]. In a previous report [39], oosporein showed antifungal activity against *Phytophthora infestans*, which causes a serious disease in solanaceous crops. Oosporein was also reported to be produced by *C*. *cupreum* isolated from a twig of *Macleaya cordata*, and oosporein shows antifungal activity against pathogenic fungi, such as *Rhizoctonia solani*, *Botrytis cinerea*, and *Pythium ultimum* [40]. Our study is the first to clarify that oosporein acts as microbial siderophore, which alleviates Al toxicity via chelation of Al in *M*. *sinensis* roots. Our results also clarified that oosporein chelated Al at a ratio of 2:1 (S4 Fig), and the stability constant for the complex between Al and oosporein was 12.1, which is an important measure to evaluate metal-detoxification strength. Siderophores, which show higher stability constants with Al, could detoxify Al more efficiently compared with those with lower stability constants [30]. Several plant species have been reported to detoxify Al using organic acids [41–43] and phenolic acids [25]. Compared with the stability constant for the complex between Al and chlorogenic acid, malic acid, or citric acid, which were detected in *M*. *sinensis* roots [9], the stability constant of oosporein-Al (12.1) was higher than those of citric acid-Al and malic acid-Al (8.0 and 5.4, respectively) [30], but lower than that of chlorogenic acid-Al (15.1) [31]; oosporein and chlorogenic acid could detoxify Al more efficiently than citric acid and malic acid.

The inoculation test using *C*. *cupreum* and *M*. *sinensis* seedlings clarified that *C*. *cupreum* increased the amount of Al in roots (Fig 5C, *P* < 0.05). In contrast, the concentration of Al in the roots was not significantly different (Fig 4C, *P* > 0.05). Compared with that of the control seedlings, *C*. *cupreum* significantly promoted seedling growth (Table 2, *P* < 0.05 and S5 Fig), and extremely quick growth of plants decreased the concentrations of inorganic elements in plants (dilution effect, [6]). Therefore, dilution of Al in plants via rapid plant growth could decrease the toxicity of Al [14, 23]. The concentrations of Al in the roots of inoculated and control seedlings were considerably high compared with other plant tissues [44–46]. This suggested that *M*. *sinensis* alone, as well as *M*. *sinensis* inoculated with *C*. *cupreum*, should detoxify high concentrations of Al; these results are consistent with a previous inoculation test using *C*. *cupreum* and *M*. *sinensis* seedlings [9].

The total amount of Al detoxified by the siderophores in the inoculation test (Table 3) showed that *C*. *cupreum* increased the Al tolerance of *M*. *sinensis* by producing oosporein and enhancing the production of chlorogenic acid, compared with the control (Fig 6). Therefore, Al detoxification with phytosiderophores and microbial siderophores would enhance Al tolerance in *M*. *sinensis* inoculated with *C*. *cupreum*. In our previous report [9], the Al localization patterns were different between the roots inoculated with *C*. *cupreum* and the control roots; Al was localized at particularly high levels in the cell walls of the epidermis, endodermis, and stele in inoculated roots. In contrast, Al was localized in cell walls throughout the control roots. This result indicated that *M*. *sinensis* could detoxify a considerable amount of Al in cell walls to inhibit Al localization in the cells. In the inoculated roots, the induction of chlorogenic-acid production and oosporein production by *C*. *cupreum* could decrease Al localization in the cell walls. This decrease might be related to plant-growth promotion, because root growth is inhibited by excessively high concentrations of Al in the cell wall [47]. In addition, the chemical enhancement of Al tolerance by *C*. *cupreum* via the production oosporein and the stimulation of chlorogenic-acid production would be important for *M*. *sinensis* to adapt to acidic mine sites.

## Conclusions

In this study, root-endophytic *C*. *cupreum* was shown to produce oosporein as a siderophore to detoxify Al. The stability constant with Al was comparatively high (12.1) at a coupling ratio 2:1. Compared with the stability constant of other Al detoxicants, oosporein could detoxify Al more efficiently than citric acid and malic acid. *Chaetomium cupreum* inoculation promoted plant growth and phytosiderophore (chlorogenic acid) production. In addition, the induction of chlorogenic-acid production and oosporein production by *C*. *cupreum* would be important in Al tolerance of *M*. *sinensis*. Our research suggests that chemical defense is a secondary factor rather than a primary factor for plants to adapt flexibly to elemental toxicity in the environment, such as acidic mine sites. We hope that the results of the present study will be helpful for the greening of mining sites.

## Supporting information

S1 Fig Culture of *Chaetomium cupreum* during an 18-day incubation. (a) pH of the culture filtrate (closed circles) and mycelial dry weight (DW) (open circles), and (b) Al-chelating activity of the culture filtrate. Results are expressed as the mean ± SE. According to the result shown in (b), a 12-day incubation time, which showed the highest Al-chelating activity, was selected for the incubation time to isolate the siderophore.
(TIF)

S2 Fig Al-chelating activity of the culture filtrate after separation. Blank, 1% malt extract liquid medium shaken for 12 days; CF, culture filtrate; O; organic layer; W, water layer; CR, crystalline powder; M, mother liquid. Results are expressed as the diameter of the clear zones (mm) ± SE. Each fraction was dried and re-dissolved in 10% methanol. Different letters indicate a statistically significant difference among treatments in one-factor ANOVA comparisons and post-hoc Tukey HDS at *P* < 0.05.
(TIF)

S3 Fig The titration curves for oosporein and the Al-oosporein complex. (a) The titration curve for oosporein. (b) The titration curve for the Al-oosporein complex.
(TIF)

S4 Fig The UV-VIS absorption spectra of Al-oosporein complex at pH 6. (TIF)

S5 Fig *Miscanthus sinensis* seedlings after the inoculation test. (a) *M*. *sinensis* seedling (control). (b) *M*. *sinensis* seedling inoculated with *Chaetomium cupreum*. Scale bar represents 10 mm.
(TIF)
