# Supplementary figures and images for: Root-endophytic Chaetomium cupreum chemically enhances aluminium tolerance in Miscanthus sinensis via increasing the aluminium detoxicants, chlorogenic acid and oosporein
Source: PLoS One. 2019 Feb 22;14(2):e0212644. doi: 10.1371/journal.pone.0212644 (PMC6386393; doi:10.1371/journal.pone.0212644)

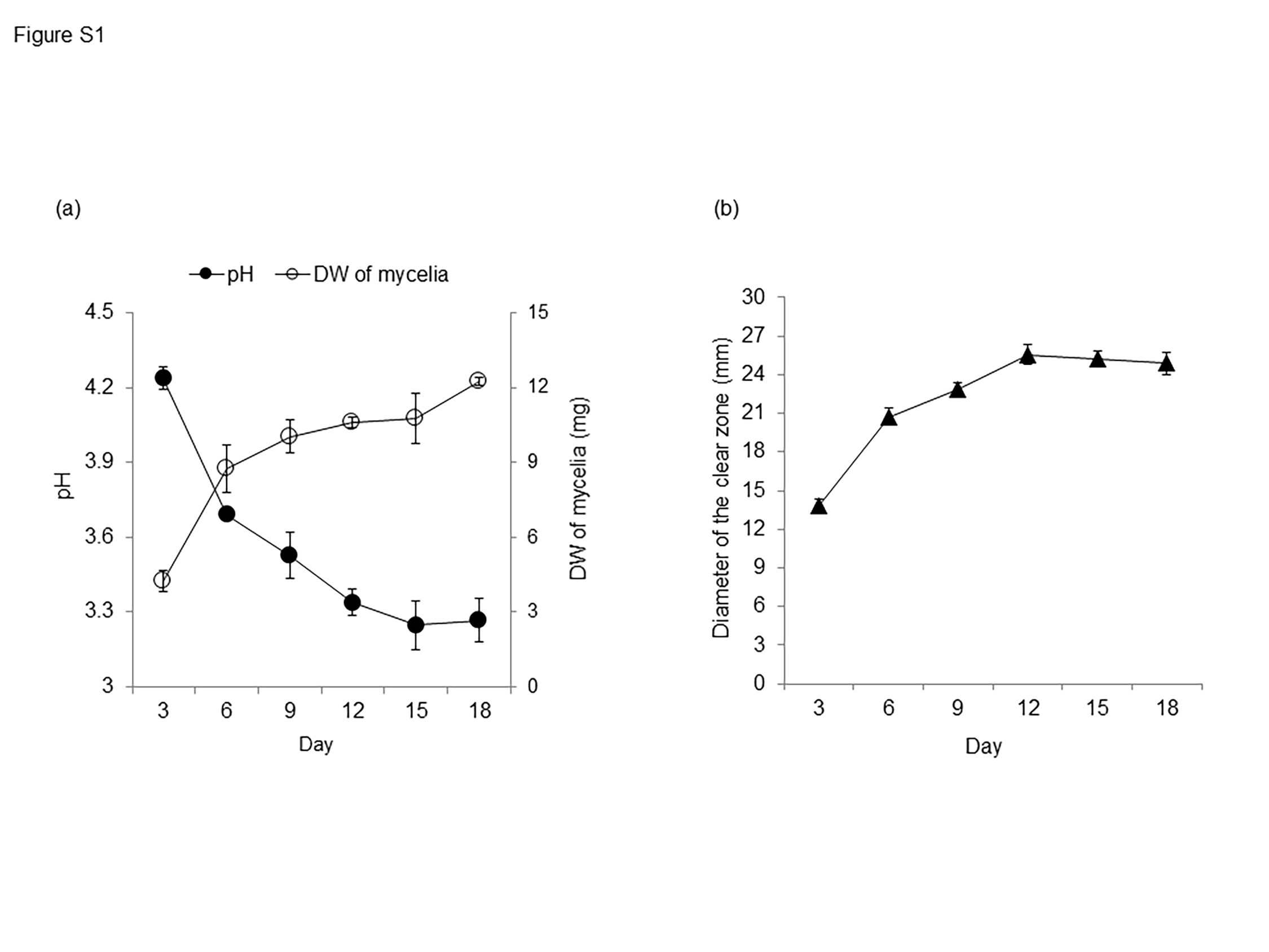

Supplement: S1 Fig — (a) pH of the culture filtrate (closed circles) and mycelial dry weight (DW) (open circles), and (b) Al-chelating activity of the culture filtrate. Results are expressed as the mean ± SE. According to the result shown in (b), a 12-day incubation time, which showed the highest Al-chelating activity, was selected for the incubation time to isolate the siderophore. (TIF) [file pone.0212644.s001.tif]

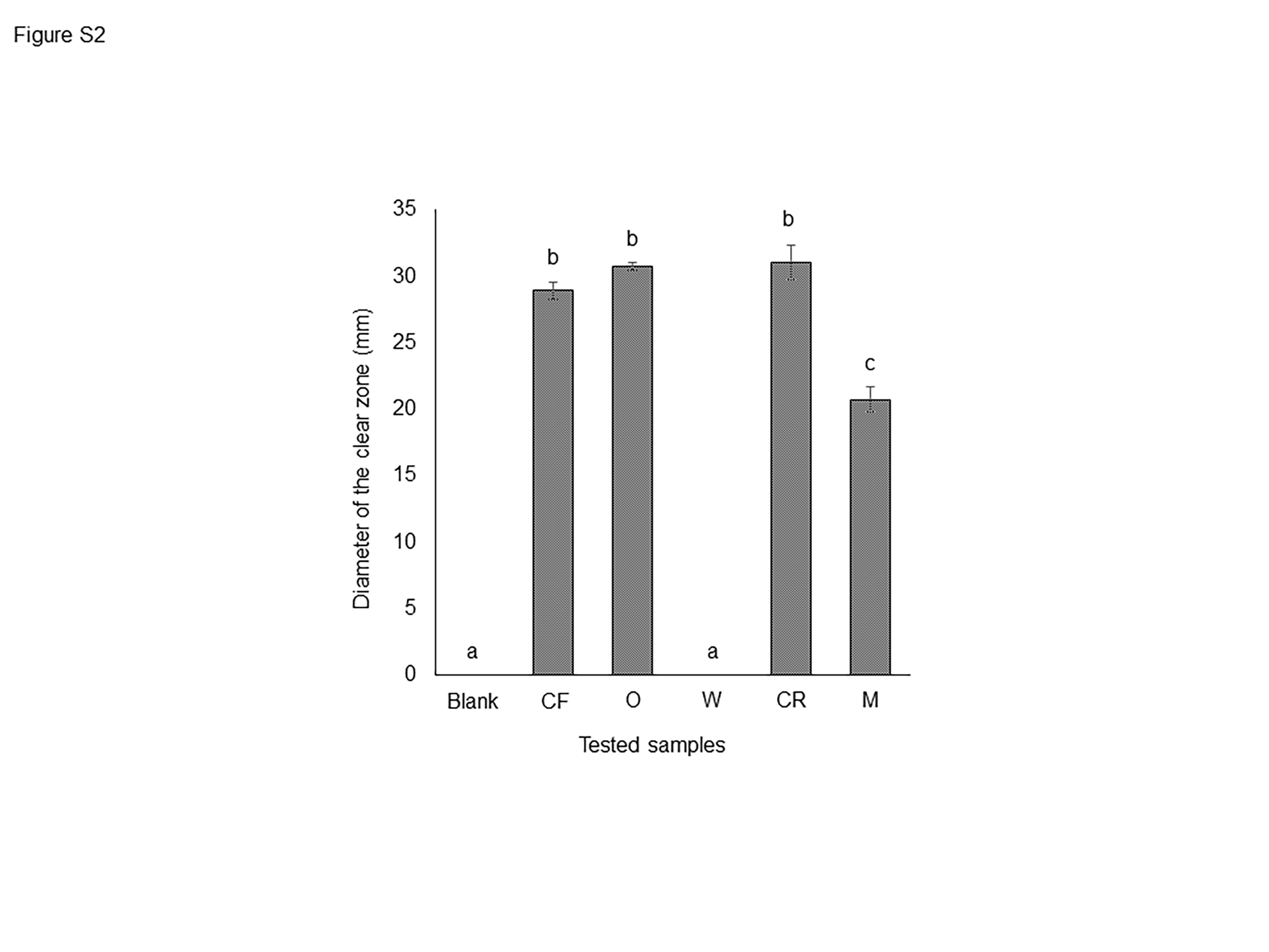

Supplement: S2 Fig — Blank, 1% malt extract liquid medium shaken for 12 days; CF, culture filtrate; O; organic layer; W, water layer; CR, crystalline powder; M, mother liquid. Results are expressed as the diameter of the clear zones (mm) ± SE. Each fraction was dried and re-dissolved in 10% methanol. Different letters indicate a statistically significant difference among treatments in one-factor ANOVA comparisons and post-hoc Tukey HDS at P < 0.05. (TIF) [file pone.0212644.s002.tif]

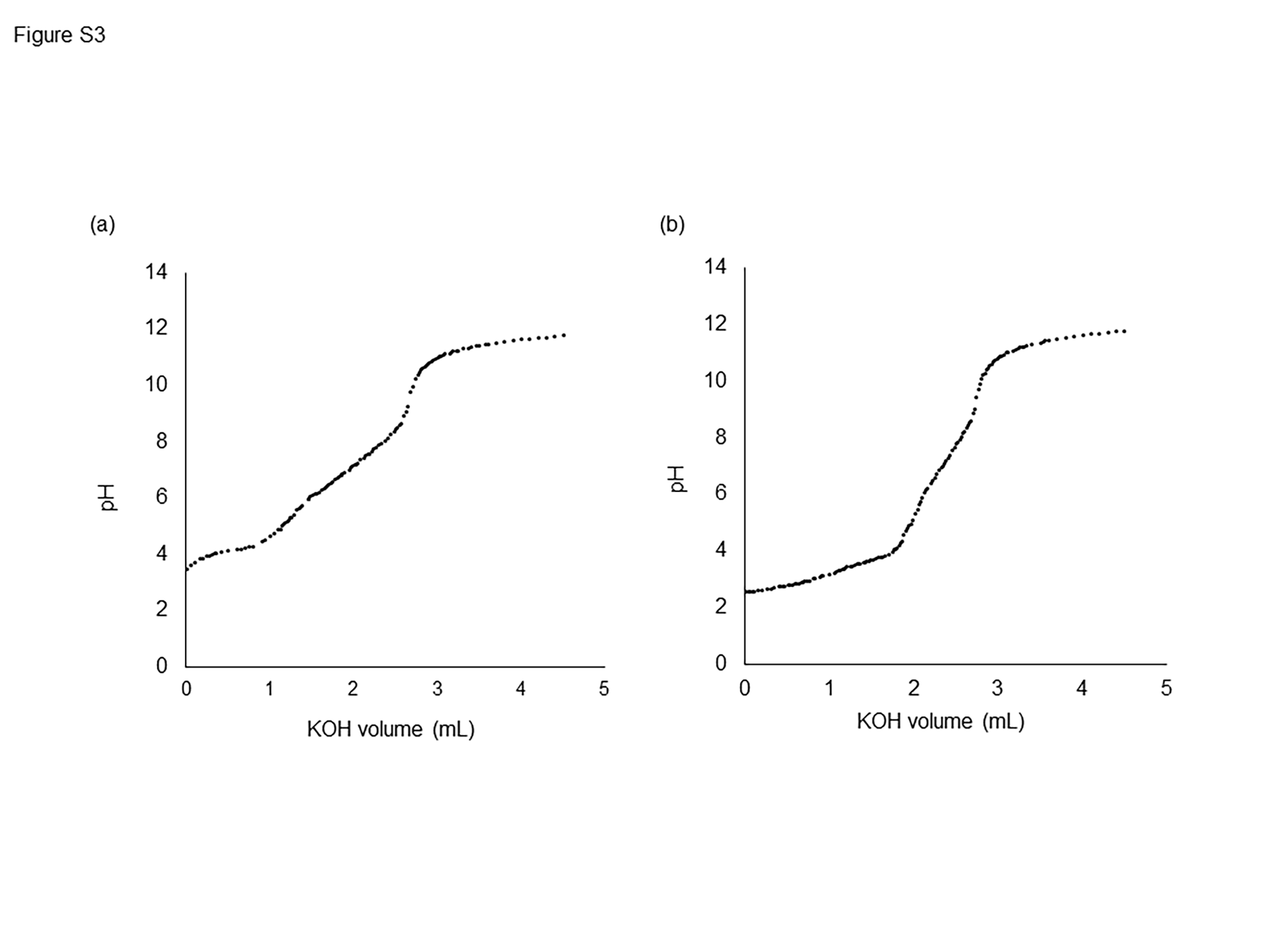

Supplement: S3 Fig — (a) The titration curve for oosporein. (b) The titration curve for the Al-oosporein complex. (TIF) [file pone.0212644.s003.tif]

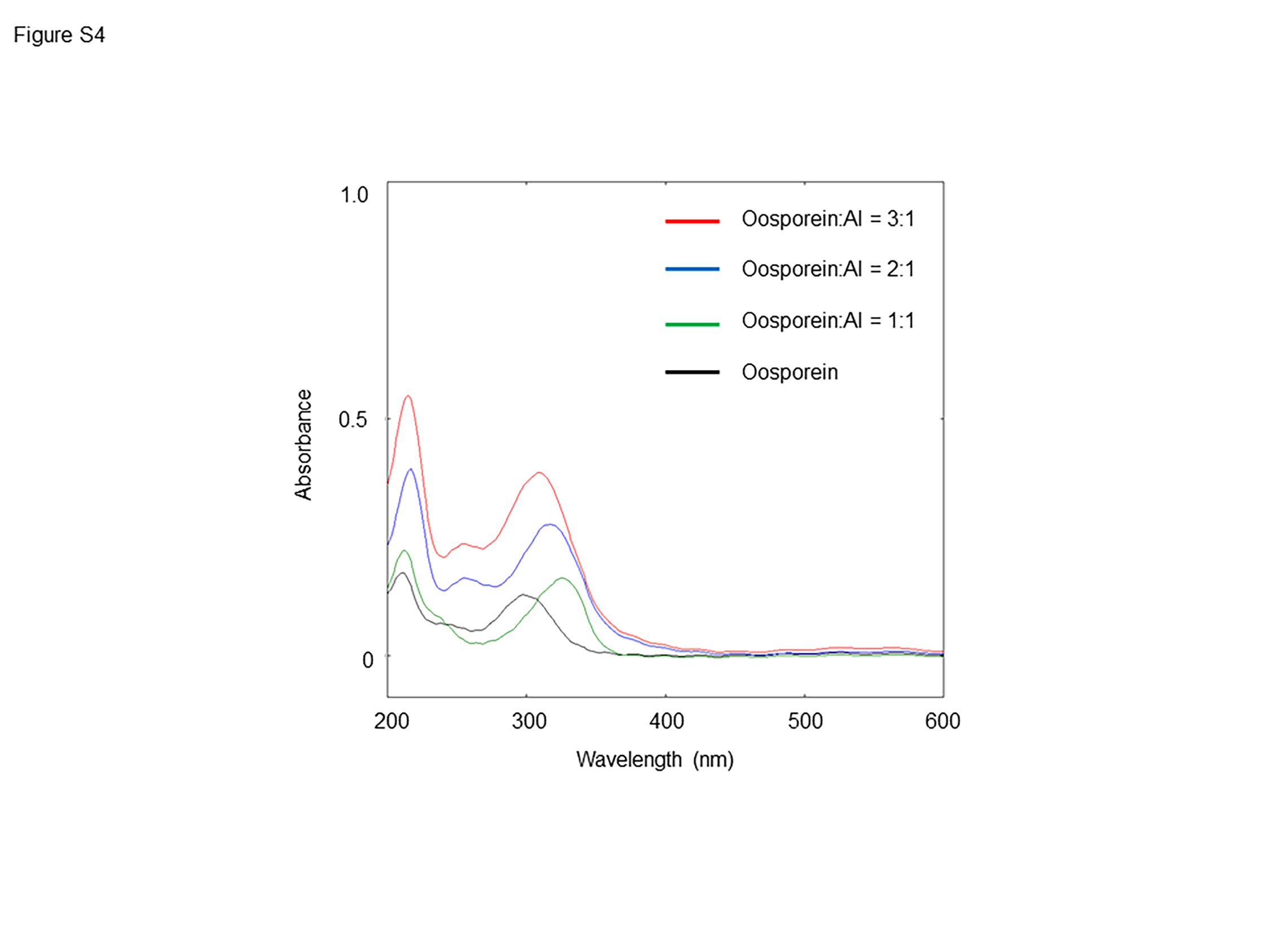

Supplement: S4 Fig — (TIF) [file pone.0212644.s004.tif]

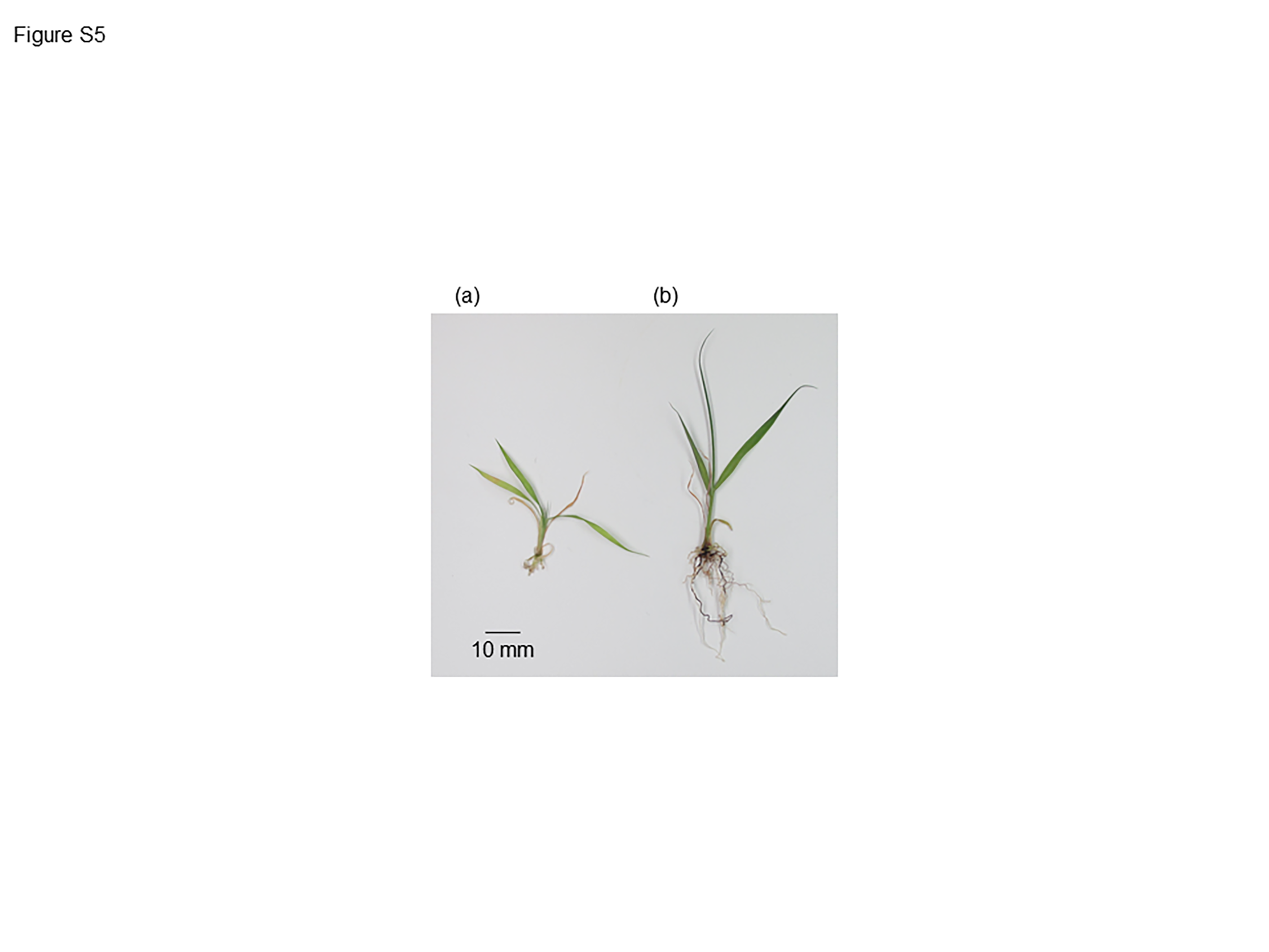

Supplement: S5 Fig — (a) M. sinensis seedling (control). (b) M. sinensis seedling inoculated with Chaetomium cupreum. Scale bar represents 10 mm. (TIF) [file pone.0212644.s005.tif]
